# Superb Microvascular Imaging (SMI) Compared with Color Doppler Ultrasound for the Assessment of Hepatic Artery in Pediatric Liver Transplants: A Feasibility Study

**DOI:** 10.3390/diagnostics12061476

**Published:** 2022-06-16

**Authors:** Elona Collaku, Roberto Simonini, Maurizio Balbi, Pietro Andrea Bonaffini, Clarissa Valle, Cesare Morzenti, Romina Fatima Faseli, Alberto Ferrari, Davide Ippolito, Paolo Marra, Tiziano Barbui, Sandro Sironi

**Affiliations:** 1 Department of Radiology, Papa Giovanni XXIII Hospital, Piazza OMS 1, 24127 Bergamo, BG, Italy; roberto.simonini89@gmail.com (R.S.); balbi.m@libero.it (M.B.); pa.bonaffini@gmail.com (P.A.B.); clarissa.valle.rad@gmail.com (C.V.); c.morzenti@asst-lecco.it (C.M.); rfaseli@asst-pg23.it (R.F.F.); pmarra@asst-pg23.it (P.M.); sandrosironi@libero.it (S.S.); 2 School of Medicine, University of Milano-Bicocca, Piazza dell’Ateneo Nuovo 1, 20126 Milan, MI, Italy; davide.atena@tiscalinet.it; 3 FROM Research Foundation, Piazza OMS 1, 24127 Bergamo, BG, Italy; aferrari34@yahoo.com (A.F.); tbarbui@fondazionefrom.it (T.B.); 4 Department of Radiology, San Gerardo Hospital, Via G. B. Pergolesi, 33, 20900 Monza, MB, Italy

**Keywords:** conventional Doppler, superb microvascular imaging, liver transplantation, pediatric, feasibility

## Abstract

(1) Background: Despite progression in surgical techniques and immunological treatments, hepatic artery (HA) thrombosis and stenosis still develop as an early or late liver transplant (LT) complication. We aimed to compare superb microvascular imaging (SMI) with conventional Doppler imaging (CDI) in the assessment of HA in a cohort of pediatric patients undergoing follow-up ultrasound (US) for LT. (2) Methods: This prospective, observational study included 73 pediatric LT recipients (median age, 7 years; IQR, 5.8 years; 35 females) who underwent US during LT follow-up from March to December 2019. For each examination, CDI and SMI were separately assessed in terms of HA visibility and spectral waveform morphology (SWM). The former was scored based on HA discrimination from the blooming signal of the surrounding vessels, as follows: 0, not visible; 1, majority course hardly distinguishable; and 2, majority course clearly distinguishable. The latter was scored on a two-point scale: 0, combined venous and arterial SWM, and 1, pure arterial SWM. The patient’s overall score was finally calculated by adding the two individual scores. (3) Results: Both the absolute scores and frequency of overall scores equal to 3 (maximum global score) were higher using SMI compared with CDI. The median overall score was 3 for SMI and 2 for CDI (*p* = 0.011; IQR = 1). An overall score equal to 3 was obtained in 74% and 49.3% of the study population using SMI and CDI, respectively (*p* = 0.002). This was attributable to a better score in HA visibility (*p* = 0.007). (4) Conclusions: SMI has shown promise for assessing HA in pediatric LT recipients, possibly serving as a complementary non-invasive tool of CDI in everyday practice.

## 1. Introduction

Despite progressive refinements in surgical techniques and immunological treatments, hepatic artery (HA) thrombosis and stenosis still occur after liver transplant (LT), in both early (up to 13% of cases) [1,2] and late (about 2.7–6.1%) phases [3,4]. These conditions require a timely diagnosis, as, if left untreated, they can lead to irreversible ischemic damage and graft failure [5].

Ultrasound (US) is the primary imaging modality for the postoperative surveillance of LT and the main tool for long-term follow-up [6,7]. It routinely entails grayscale assessment of the liver parenchyma and biliary tree and Doppler evaluation (hereafter called conventional Doppler imaging (CDI)) of the venous and arterial hepatic vasculature [7]. Although the US enables the identification of HA complications with a high diagnostic accuracy [8,9,10], it may be inconclusive in cases of periportal edema, vasospasm, low cardiac output, and small-size HA [11]. Contrast-enhanced US (CEUS) has the potential to overcome CDI limitations, allowing for assessment of the liver microvasculature and perfusion kinetics. Nevertheless, it lacks approval for use in pediatric patients [12]. When the HA is not properly visualized with US, computed tomography angiography (CTA) is required, but there are rising concerns of radiation exposure and iodine contrast media in the pediatric population [13].

Superb microvascular imaging (SMI) is a recently developed US technique based on an adaptive algorithm capable of separating low flow signals from overlaying tissue motion artifacts, thereby enabling visualization of microscopic vessels without employing contrast agents [14,15].

On these bases, our study aimed to explore the potential of SMI in visualizing HA in pediatric LT recipients compared with CDI, which was used as an imaging standard of reference.

## 2. Materials and Methods

The Independent Ethics Committee of Bergamo approved this prospective, observational, single-center study (protocol code 2018-0176). Written informed consent was obtained from all of the study participants’ parents or legal guardians.

Study Population

Between March and December 2019, 75 consecutive pediatric LT recipients who underwent routine abdominal US at the Radiology Department of a single referral center for pediatric LT were prospectively enrolled. The inclusion criteria were as follows: (a) prior orthotopic LT, (b) US referral for routine LT monitoring or suspicion of LT complication(s), and (c) age <18 years old. The exclusion criteria were as follows: (a) LT performed within 1 month before US and (b) inadequate image quality due to non-cooperative patients.

US protocol

All examinations were performed using a Toshiba Aplio 500 US scanner (Canon Medical Systems Corporation; Tustin, CA, USA), with a curvilinear 3.5 MHz transducer (PVT-375BT). The scan parameters were set to default in the abdominal examination protocol and were then individually optimized by the operator during the scan. Each patient was first evaluated by the consultant radiologist in charge (with at least 5 years of experience in pediatric abdominal US imaging), who performed CDI after a grayscale evaluation of the upper abdomen, as per routine protocol in our institution. SMI was then performed in the same examination session by a 10-year experienced radiologist in abdominal US (CM) blinded to CDI results. All of the SMI views were imaged in monochrome mode only.

Study endpoints

The examiners provided a real-time, qualitative evaluation of HA visibility and spectral wave morphology (SWM) both for CDI and SMI. The HA visibility was categorized as not visible (0 points), hardly distinguishable (1 point), or clearly distinguishable (2 points). The HA SWM was classified as pure arterial (1 point) or admixed arterial and venous (0 points). If the HA was not visible, SWM was scored as 0. The final patient score was computed as the sum of the two scores (i.e., ranging from 0 to 3). The HA resistive indexes were calculated by both examiners in all cases. A routine radiology report was generated for all of the participants.

Statistical analysis

Statistical analysis was performed using Stata software version 13 (StataCorp.2013. Stata Statistical Software: Release 13. College Station, TX: StataCorp LP, USA). HA visibility (i.e., frequency of final US scores equal to 3) was compared between SMI and CDI using an exact symmetry test. Scores from SMI were compared with those from the CDI by Wilcoxon sign-rank test. In all of the tests, statistical significance was considered for *p* < 0.05.

## 3. Results

After the exclusion of two patients due to poor compliance during the examination, a total of 73 patients constituted the final sample of our analysis.

The demographic and clinical features of the study participants are listed in Table 1. Most patients were male (*n* = 38/73, 52%). The median age was 7 years old (IRQ = 5.8 years). The most frequent cause of LT was biliary atresia (*n* = 44/73, 60%), followed by cryptogenic primary cirrhosis (*n* = 8/73, 11%). A total of 50 patients (69%) had a previous early complication after LT: acute rejection, HA stenosis, biliary fistula, biliary anastomosis strictures or dehiscence, acute cholangitis, portal vein thrombosis, and cytomegalovirus infection. Resistive indexes (RI) were within the range of normality (between 0.5 and 0.8) in all cases [10,11,16].

**Figure 1:**
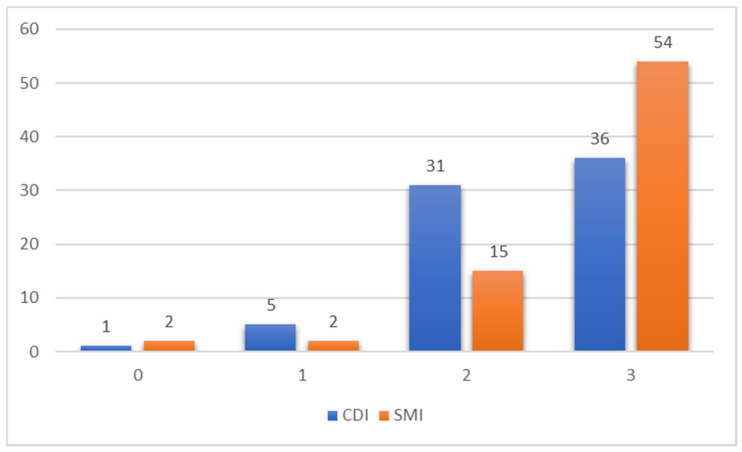
Graphic representation of the total score values, from 0 to 3 points of CDI and SMI evaluation. CDI—conventional Doppler imaging; SMI—superb microvascular imaging.

**Figure 2:**
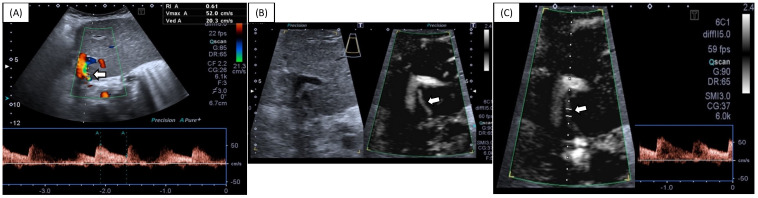
A 10 year old female undergoing standard abdominal US follow-up for prior LT. HA assessment is performed with CDI (**A**) and SMI (**B**,**C**). Visualization and sampling of the HA (arrows) are comparable in this case. Arrows indicate HA. HA—hepatic artery; CDI—conventional Doppler imaging; SMI—superb microvascular imaging.

**Figure 3:**
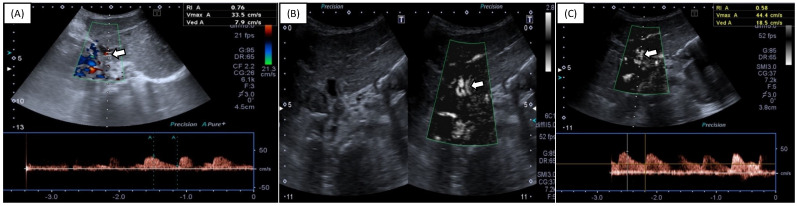
A one-year-old female undergoing abdominal US follow-up for LT, with standard CDI (**A**) and SMI (**B**,**C**). SMI allowed for improved HA visualization and sampling over CDI, with a more definite and stable waveform (**C**). Arrows indicate HA. HA—hepatic artery; CDI—conventional Doppler imaging; SMI—superb microvascular imaging.

Table 2 and Figure 1 show score comparisons after US evaluation using SMI vs. CDI. Both CDI and SMI had a median HA visibility score equal to 2, with an interquartile range of 1 for CDI and 0 for SMI (*p* = 0.007). No significant differences were observed in terms of SWM visualization between SMI and CDI (example in Figure 2), although SMI performed slightly better in some cases (example in Figure 3): an admixed arterial and venous SWM (lowest score of the variable) was observed in five patients during SMI evaluation (7%) and in seven patients during CDI evaluation (10%) (*p* = 0.765). No patients underwent further imaging investigations based on the US results.

## 4. Discussion

Compared with adult patients, pediatric liver recipients are at higher risk of developing HA thrombosis due to technical difficulties presented by smaller blood vessels and size mismatch between graft and recipient vessels. Moreover, HA is obscured by portal venous flow during US examination [1], or it is difficult to evaluate because of periportal edema, vasospasm, low cardiac output, and small HA size [12]. Therefore, it would be useful to implement a non-invasive tool able to complement CDI and enhance the accuracy of abdominal US in the assessment of HA during LT follow-up.

SMI relies on an algorithm that suppresses visual noise without subtracting low-velocity flow. The technique uses an adaptive wall filter different from CDI and minimizes flash artifacts. Even without using any contrast medium, SMI can improve the sensitivity of slow-flow visibility and the signal detection of small blood vessels [17]. Additionally, SMI employs a short pulse and high frame rates (>50 frames/s), resulting in improvements in distance resolution and reduced blooming. Therefore, it may be helpful in settings where the blooming signal from the portal vein obscures the signal from the adjacent HA, which is a technical pitfall that may occur using CDI [17,18].

SMI has been tested to assess a broad spectrum of disorders, including breast tumors, thyroid nodules, carotid plaques, vascular endoleak, and focal liver lesions [19,20,21,22,23,24,25,26,27,28]. However, there are still a few studies regarding SMI technique application during the routine follow-up of LT patients [18,29], particularly in pediatric recipients [17]. Therefore, we aimed to explore the potential of SMI in visualizing HA in pediatric LT recipients compared with CDI.

The current findings show that both the absolute score and frequency of the maximum global scores were higher when using SMI than CDI because of the higher SMI scoring in HA detection but not SWM visualization. Our study suggests that SMI may have the potential to improve HA visibility and complement CDI when evaluating pediatric liver recipients in daily clinical practice. This benefit is attractive, especially for less confident operators who may improve their diagnostic performance by employing such a non-invasive tool. However, we did not perform analyses to properly evaluate the SMI added value among readers of different expertise, which warrants future studies.

Li-hong Gu et al. [17] recently reported that SMI can show the distribution of hepatic arterial blood flow and can provide more details than CDI alone, markedly improving the visualization of the HA after pediatric LT, in line with our results. Their study population consisted of pediatric patients evaluated after LT during intensive care unit monitoring. Our work differs from that one as we evaluated pediatric patients at least 30 days after surgery, a setting that, to the best of our knowledge, has not been explored in the literature so far.

Our findings align with previous studies that explored SMI for HA assessment in adult liver recipients. Jang et al. [29] compared SMI and CDI in LT patients. They concluded that SMI shows good reproducibility, correlates well with currently used methods for postoperative evaluation of HA in LT recipients, and is further improved by administering a US contrast agent. Similarly, Güven et al. [18] investigated the sensitivity and specificity of SMI in the assessment of HA occlusion after LT in a cohort of 95 adult patients who underwent LT from living or cadaver donors. The authors concluded that SMI could improve the confidence of US in HA occlusion diagnosis without the need for invasive techniques in patients with suspected thrombosis at CDI. The main drawback of US-related techniques in adults is the lack of panoramic view and the potentially limited acoustic window; these technical concerns are less important when dealing with pediatric patients.

The present study has some limitations. First, in some cases, it may be difficult to perform US in pediatric patients due to their limited compliance. To some degree, this aspect may account for the proportion of scores that did not reach the maximum value with SMI. The lack of HA thrombosis cases in our study population did not allow for evaluation of SMI in terms of diagnostic performance; therefore, we focused on the technical feasibility. We did not make a time estimation for HA waveform acquisition during SMI compared with CDI. However, our preliminary observations possibly argue in favor of SMI to shorten the whole US scan timing by improving HA visualization. Furthermore, we did not compare SMI with CEUS. However, CEUS involves an intravenous contrast medium that has not been approved yet in Europe for intravascular use in pediatric patients. Finally, none of our patients underwent further imaging investigations based on the US results, which would have confirmed or contradicted the SMI findings.

In conclusion, SMI has shown promise for assessing HA in pediatric LT recipients, possibly serving as a complementary non-invasive tool of CDI in daily practice. Further studies are needed to explore the diagnostic performance of SMI compared with CDI and to understand its potential to avoid unnecessary CT examination when HA complications are suspected.

## Figures and Tables

**Table 1:** Study population characteristics.

Variable	Value
Total population *n*	73
Age (years) Median (IQR)	7 (5.8)
Sex *n (%)*	
F	35 (47.9)
M	38 (52.1)
Weight (kg) Median (IQR)	19.95 (19)
Underlying liver disease *n (%)*	
Biliary atresia	44 (60.3)
Cryptogenic primary cirrhosis	8 (11)
Alagille syndrome	6 (8.2)
Familial cholestasis	4 (5.5)
Neoplasms	4 (5.5)
Other	7 (10)
Acute postoperative complications *n (%)*	50 (68.5)

IQR—interquartile range.

**Table 2:** Score comparison.

Parameter		CDI	SMI	*p*
HA visibility score (0 to 2 points) Median (IQR)		2.0 (1.00)	2.0 (0.00)	0.007 **
			
			
SWM (0 to 1 point) *n* (%), total 73 patients	Score 1	66 (90)	68 (93)	
	Score 0	7 (10)	5 (7)	0.765
Total score (0 to 3 points) Median (IQR)		2.00 (1.00)	3.00 (1.00)	0.011 *
Total score < 3 *n* (%)		37 (50.7)	19 (26.0)	0.002 **
Total score = 3 *n* (%)		36 (49.3)	54 (74.0)	

* Score comparison between CDI and SMI by method. Exact symmetry test on frequencies, Wilcoxon signed-rank test on raw scores. * *p* < 0.05, ** *p* < 0.01. CDI—conventional Doppler imaging; SMI—superb microvascular imaging; HA—hepatic artery; SWM—spectral waveform morphology; IQR—interquartile range.

## Data Availability

Not applicable.
